# Contribution of informal caregivers to self-care in individuals with heart failure

**DOI:** 10.1590/0034-7167-2023-0492

**Published:** 2024-07-29

**Authors:** Mailson Marques de Sousa, Adriana Meira Tiburtino Nepomuceno, Rayana Pereira Feitosa, Lara de Sá Neves Loureiro, Renan Alves Silva, Maria das Graças Melo Fernandes, Simone Helena dos Santos Oliveira

**Affiliations:** I Universidade Federal da Paraíba. João Pessoa, Paraíba, Brazil; II Hospital Universitário Lauro Wanderley. João Pessoa, Paraíba, Brazil; III Universidade Federal de Campina Grande. Cajazeiras, Paraíba, Brazil

**Keywords:** Heart Failure, Self-Care, Nursing, Caregivers, Cardiology, Insuficiencia Cardíaca, Autocuidado, Cuidadores, Enfermería, Cardiología

## Abstract

**Objectives:**

to evaluate the contribution of informal caregivers to the self-care of individuals with heart failure.

**Methods:**

a cross-sectional study was conducted with 87 caregivers from March to October 2022 in the city of João Pessoa/PB. The caregivers’ contribution was assessed using the Caregiver Contribution to Self-Care of Heart Failure Index instrument. Scores ≥ 70 points indicate adequate contribution. Data were analyzed using descriptive statistics and Spearman’s correlation.

**Results:**

the sample consisted of 81.6% female caregivers. Median scores obtained for the self-care contribution scales were: 63.3 for maintenance; 55.5 for management; and 66.6 for confidence. Caregivers never or rarely recommended monitoring body weight, regular physical exercise, extra use of diuretics, and fluid restriction.

**Conclusions:**

informal caregivers showed inadequate contribution in the areas of maintenance, management, and confidence in self-care of individuals with heart failure.

## RESUMEN

**Objetivos:**

evaluar la contribución de cuidadores informales para el autocuidado de personas con insuficiencia cardíaca.

**Métodos:**

estudio transversal, realizado con 87 cuidadores, de marzo a octubre de 2022, en la ciudad João Pessoa/PB. La contribución del cuidador fue evaluada mediante el instrumento *Caregiver Contribution to Self-Care of Heart Failure Index*. Escores ≥ 70 puntos indican contribución adecuada. Los datos fueron analizados mediante estadística descriptiva y correlación de Spearman.

**Resultados:**

son del sexo femenino 81,6% de la muestra. La mediana de los escores obtenidos para las escalas de contribución para el autocuidado fueron: 63,3 para manutención; 55,5 para manejo; y 66,6 para confianza. Los cuidadores nunca o raramente recomendaban el monitoreo del peso corporal, la práctica regular de ejercicios físicos, el uso extra de diuréticos y la restricción de líquidos.

**Conclusiones:**

los cuidadores informales presentaron contribución inadecuada en los quesitos manutención, manejo y confianza del autocuidado de personas con insuficiencia cardíaca.

## INTRODUCTION

Heart failure (HF) is a chronic disease associated with significant morbidity and mortality rates, low quality of life, and high financial costs for health systems^(1)^. It is estimated that 64.34 million people worldwide have HF^(2)^. Projections indicate that the prevalence of HF in the United States is expected to increase from 5.8 million to 8.5 million by 2030^(3)^. In Brazil, during the triennium 2019-2021, 533,055 hospital admissions for individuals with HF were authorized just within the Unified Health System (SUS), with a mortality rate of 12.27% for the period^(4)^.

Self-care (SC) constitutes a fundamental strategy for the therapeutic management of HF. It involves a naturalistic decision-making process that includes the choice of behaviors taken by patients to maintain physiological stability and respond to symptoms of HF exacerbation when they occur^(5)^. Better levels of self-care are related to lower readmission rates, improved quality of life, and reduced mortality^(6-7)^.

Studies show that SC behaviors in individuals with HF are unsatisfactory^(8-9)^. Factors such as knowledge, attitudes, skills, motivation, beliefs, cultural differences, and access to health services are associated^(5)^. SC behaviors in individuals with HF are often developed with the contribution of informal caregivers (unpaid individuals with whom the patient has a pre-established relationship, such as spouses, children, family members, or friends)^(10)^. About 45% to 70% of patients with HF live with a family member involved in their care^(11)^.

In HF, the caregiver’s contribution to SC is defined as the process through which caregivers support patients in maintaining disease stability, monitoring, perceiving, and managing signs and symptoms, mediated by confidence in contributing to the patients’ SC under their care^(10)^. Thus, informal caregivers assist by recommending the person with HF to check their daily body weight, consume a low-sodium diet, control fluid intake, use prescribed medications, practice physical activities, and undergo vaccination. They provide social support in symptom monitoring and responses to clinical manifestations related to the disease when they occur. This help includes making time, effort, and support available on behalf of another person who is performing SC in daily life actions and assistance in decision-making^(10-12)^.

An integrative review study^(13)^ found that most research assessing the caregiver’s contribution to SC was developed in Europe and the Middle East. In South America, there is a lack of evidence, and the practices carried out by caregivers in favor of SC in this population are not clear. In Brazil, a study conducted in the Southeast Region^(14)^ highlighted inadequate contribution. To date, no studies on this theme have been found in the Brazilian Northeast. The necessity for new research to understand the phenomenon and to verify the caregiver’s contribution to decision-making for self care and the management of heart failure is evident. Such studies are crucial for minimizing unwanted hospitalizations, improving health-related quality of life, and designing feasible educational interventions in clinical practice.

## OBJECTIVES

To evaluate the contribution of informal caregivers to the self-care of individuals with heart failure.

## METHODS

### Ethical Aspects

The Research Ethics Committee of the proposing institution approved the research project. Participants received verbal and written information about the study and formalized their participation by signing the Informed Consent Form (ICF).

### Design, Period, and Place of Study

This is a cross-sectional study in which the recommendations of the Strengthening the Reporting of Observational Studies in Epidemiology (STROBE) were followed. It was developed in a cardiology outpatient clinic of a university hospital affiliated with the Unified Health System (SUS), a reference for medium and high complexity cardiology care in the city of João Pessoa, state of Paraíba, Brazil. In this outpatient clinic, the municipal health network for specialized cardiology consultations, which occur weekly, refers individuals with HF. At the selected institution, there are neither nursing consultations nor support groups established under a follow-up protocol for people with HF. However, caregivers accompany the consultations of individuals with HF.

### Population or Sample; Inclusion, and Exclusion Criteria

The study population consisted of informal caregivers accompanying individuals with HF in outpatient follow-up at the institution. The non-probabilistic sample was selected for convenience during the data collection period between March and October 2022; it consisted of 87 caregivers designated by patients with HF as their main reference for care.

Regarding eligibility criteria, informal caregivers aged ≥ 18 years who had been caring for the patients for at least three months were included. This three-month period was considered based on a previous study^(15)^ that identified an evolution in self-care actions in individuals with HF. Exclusion criteria were: caregivers who had difficulty understanding the study’s objective or answering the data collection instruments and paid caregivers.

### Study Protocol

Data were collected through structured individual interviews in a private setting, with each interview averaging 30 minutes. A research team composed of three nurses with clinical experience in cardiology was trained and attended the outpatient clinic, equipped with a list of patients scheduled for consultation that day. This list was extracted from the internal scheduling system to identify those patients who had responsible caregivers and those who met the inclusion criteria. Thus, before or after the medical consultation, caregivers were invited to participate in the research. All those who met the inclusion criteria and were invited accepted the invitation.

The following instruments were used for data collection: a) a caregiver characterization form containing certain variables (age, gender, self-declared skin color, marital status, education, family income, work situation, the caregiver’s relationship with the patient, daily hours of care, attendance at consultations, and participation in health education activities); b) Caregiver Contribution to Self-Care of Heart Failure Index (CC-SCHFI) translated and validated version for Brazilian Portuguese to assess the caregiver’s contribution to SC in HF^(16)^.

The CC-SCHFI consists of 22 items distributed in three scales: Caregiver’s Contribution to SC Maintenance-ten items (evaluates caregiver behaviors for symptom monitoring and adherence to therapeutic measures for maintaining HF stability); Caregiver’s Contribution to SC Management-six items (measures the caregiver behaviors adopted to recognize decompensation symptoms and intervene in HF symptoms); and Caregiver’s Confidence to Contribute to SC-six items (analyzes the caregiver’s ability to encourage the patient to perform SC)^(16)^. The authors of the CC-SCHFI authorized the use of the instrument, which showed adequate evidence of validity and reliability in the Brazilian population^(16)^. In this investigation, the reliability index of the CC-SCHFI scales assessed through Cronbach’s alpha was 0.73 for the SC maintenance scale; 0.61 for the SC management scale; and 0.88 for the SC confidence scale.

The CC-SCHFI’s response options vary on a four-point Likert scale for each of the scales. For the SC maintenance scale, the responses range from “never/rarely” to “always/daily”; on the SC management scale, they vary from “unlikely” to “very likely”; and for the caregiver’s confidence scale to contribute to SC, the responses go from “no confidence” to “extreme confidence”. The standardized total score ranges from 0 to 100 and each subscale must be calculated and analyzed separately. Scores ≥ 70 points indicate an adequate caregiver contribution to SC^(16)^.

### Analysis of Results and Statistics

Data were organized in an Excel spreadsheet and then transferred to the Statistical Package for the Social Sciences (SPSS) software version 22.0. The normality of the data was assessed using the Kolmogorov-Smirnov test, which identified an asymmetrical distribution. Data were analyzed using descriptive statistics. Categorical variables were expressed in absolute and relative frequencies, while numerical variables were described using medians and interquartile ranges 25 and 75.

The Spearman correlation coefficient analysis was employed between the CC-SCHFI scores and quantitative variables of interest (age, education, and hours of care) based on literature evidence^(10,17)^ highlighting these caregiver characteristics as possible factors influencing the contribution to SC in HF. To classify the strength of the correlations, the following parameter was adopted: < 0.30 weak magnitude; ≥ 0.30 and ≤ 0.50 moderate magnitude; > 0.50 strong magnitude^(18)^. The significance level adopted for the analyses was *p* < 0.05.

## RESULTS

Regarding the profile of informal caregivers (n = 87), 81.6% were female, with a median age of 46 years, 56.3% were of mixed race, 71.3% were married, 29.9% were self-employed, 56.3% had a family income of one minimum wage, and the median education level was 8 years. Regarding the relationship of the caregiver to the patient, 75.9% were family members, with 41.4% being daughters and 34.5% being wives; 24.1% had no familial relationship (neighbors/friends), 62.1% lived with the patient, providing a median of 12 hours of daily care, and 89.7% did not participate in educational activities related to SC in heart failure.

Table 1 presents the description of caregivers’ contribution scores to self-care in HF. None of the CC-SCHFI scales reached the cut-off point ≥ 70 points considered as an adequate contribution to SC.

**Table 1:** Caregiver Contribution to Self-Care of Heart Failure Index Scores, João Pessoa, Paraíba, Brazil, 2022

Scales	Median	IQ25-75^*^	Minimum-Maximum
SC Maintenance	63.3	53.3-73.3	17-100
SC Management	55.5	44.4-72.2	11-89
SC Confidence	66.6	55.5-77.7	11-100

* *Interquartile Range (25-75); SC - self-care.*

Table 2 presents the frequency of responses by item for self-care (SC) maintenance from the CC-SCHFI. When considering the contributions of informal caregivers, they never or rarely recommended the individual with HF to weigh themselves daily (55.2%), engage in some physical activity (42.5%), and exercise for 30 minutes (43.7%). However, they supported them in attending appointments with doctors or nurses (73.6%) and using reminder systems for taking medications (42.5%).

**Table 2:** Response Frequencies for Self-Care Maintenance Items, João Pessoa, Paraíba, Brazil, 2022

Self-Care Maintenance	*Never or Rarely*	*Sometimes*	*Frequently*	*Always or Daily*
Weighing daily	48 (55.2)	20 (23.0)	14 (16.1)	5 (5.7)
Checking if ankles are swollen	13 (14.9)	15 (17.2)	37 (42.5)	22 (25.3)
Avoiding getting sick (e.g., getting vaccinated against the flu and avoiding contact with sick people)	-	9 (10.3)	24 (27.6)	54 (62.1)
Engaging in some physical activity	37 (42.5)	16 (18.4)	21 (24.1)	13 (14.9)
Attending appointments with a doctor or nurse	-	6 (6.9)	17 (19.5)	64 (73.6)
Consuming a low-salt diet	4 (4.6)	8 (9.2)	17 (19.5)	58 (66.7)
Exercising for 30 minutes	38 (43.7)	16 (18.4)	21 (24.1)	12 (13.8)
Remembering to take medications	4 (4.6)	11 (12.6)	16 (18.4)	56 (64.4)
Requesting low-salt food when eating out or visiting someone	5 (5.7)	9 (10.3)	32 (36.8)	41 (47.1)
Using a system (pillbox, reminders, etc.) to help remember medications	21 (24.1)	14 (16.1)	15 (17.2)	37 (42.5)

SC - self-care.

In the management of SC, informal caregivers reported it was unlikely to recommend taking an extra diuretic (89.7%) or reducing fluid intake (31.0%). The majority of caregivers recommended reducing salt in the diet (63.2%) and immediately contacting a doctor or nurse to request therapeutic and/or care guidance (79.3%) (Table 3).

**Table 3:** Response Frequencies for Self-Care Management Items, João Pessoa, Paraíba, Brazil, 2022

SC Management	*Did not recognize*	*Took a while/Took some time*	*Quickly*	*Immediately*
If the person you are caring for has difficulty breathing or swelling in the ankles... How quickly did you recognize this as a symptom of heart failure?	12 (13.8)	24 (27.6)	30 (34.5)	21 (24.1)
Reducing salt in the diet	*Unlikely* 4 (4.6)	*Somewhat likely* 4 (4.6)	*Likely* 24 (27.6)	*Very likely* 55 (63.2)
Reduzir a ingestão de líquidos	27 (31.0)	12 (13.8)	18 (20.7)	30 (34.5)
Taking an extra diuretic	78 (89.7)	4 (4.6)	2 (2.3)	3 (3.4)
Contacting the doctor or nurse for guidance	-	-	18 (20.7)	69 (79.3)
Think about a measure you tried the last time the person you care for had difficulty breathing or swelling in the ankles... How sure were you that this measure helped?	*Did not try/None* 8 (9.2)	*Somewhat sure* 17 (19.5)	*Sure* 42 (48.3)	*Very sure* 20 (23.0)

*SC - self-care.*

Regarding confidence in contributing to SC, caregivers felt much or extreme confidence in following the treatment and less confidence in doing something to alleviate the symptoms of HF or recognizing health changes (Table 4).

**Table 4:** Response Frequencies for Confidence in Contributing to Self-Care, João Pessoa, Paraíba, Brazil, 2022

Confidence in Contributing to SC	*None*	*A little*	*Much*	*Extreme*
Preventing HF symptoms	6 (6.9)	14 (16.1)	50 (57.5)	17 (19.5)
Following treatment recommendations	1 (1.1)	6 (6.9)	49 (56.3)	31 (35.6)
Evaluating the importance of HF symptoms	6 (6.9)	14 (16.1)	46 (52.9)	21 (24.1)
Recognizing health changes in the person you care for	3 (3.4)	11 (12.6)	46 (52.9)	27 (31.0)
Doing something that alleviates HF symptoms	5 (5.7)	20 (23.0)	45 (51.7)	17 (19.5)
Evaluating how much a measure works	5 (5.7)	18 (20.7)	46 (52.9)	18 (20.7)

*SC - self-care.*

Table 5 presents the Spearman correlation coefficients between the CC-SCHFI scores and caregiver variables. There were correlations of weak magnitude and not significant.

**Table 5:** Spearman Correlation Coefficients between Caregiver Variables and Caregiver Contribution to Self-Care of Heart Failure Index Scales, João Pessoa, Paraíba, Brazil, 2022

Variables	SC Maintenance	SC Management	SC Confidence
Age	-0.09	-0.14	0.01
Education	0.19	0.20	0.17
Hours of care	-0.02	0.00	0.06

*SC - self-care.*

## DISCUSSION

This study aimed to evaluate the caregiver’s contribution to self-care in individuals with HF in a capital of the Northeast of Brazil. Regarding the profile of informal caregivers, a prevalence of women was observed, similar to what was found in an American^(19)^ and a national study^(16)^. This data corroborates the fact that society’s gender roles assign women sociocultural roles of family protection, with responsibilities for carrying out daily life activities and personal and emotional care, indicating their significant function in caring for people, especially within the family context. Additionally, the caregivers interviewed had a median age lower than observed in other studies^(19-20)^.

The low level of education and absence of participation in educational activities by the caregivers in this study are conditions that may interfere with the caregiver’s contribution to SC. These results are similar to those identified in a qualitative study^(21)^ that highlighted, among the challenges faced by caregivers in caring for individuals with HF, the deficit in basic knowledge about the disease and medications, as well as a lack of adequate guidance from health team professionals and health service organization. Another study observed that caregivers provided care with uncertainty and inadequate knowledge^(13)^. Likewise, the findings of the present research reinforce the need for the reference team to enable health education strategies for the caregiver and incorporate them into clinical care to promote the development of skills for performing safe care.

In this investigation, although the majority of caregivers were family members, likely the knowledge about the syndrome, the skills, and the time dedicated to care were insufficient to favor the maintenance of SC. HF is a chronic disease with a complex therapeutic regimen that requires engagement in lifestyle changes and continuous social support to incorporate SC actions^(5,7)^. Research indicates that overload, stress, and symptoms of anxiety and depression impair the caregiver’s contribution to SC^(10,12-13)^. Given this finding, it is recommended to develop new studies to explore the degree of the patient-caregiver relationship and its impact on SC behavior.

It was observed that the contribution to SC given by the caregivers was inadequate, as none of the scales (maintenance, management, and confidence in SC) reached the cutoff point ≥ 70 points. The scores obtained were higher than those found in a study conducted in Italy^(17)^ and similar to those from a study carried out in São Paulo^(14)^. A possible explanation is that caregivers need directed guidance, time, social support, and motivation for the exercise of activities, especially for the non pharmacological management of the person with HF. Another aspect to consider is the absence of specific nursing consultations in the follow-up of this population in the local context. These data are relevant for planning improvements in health services in terms of prior orientations and usual care provided by the multidisciplinary team.

In the descriptive analysis of the items that comprise the CC-SCHFI in the scale of caregiver contribution to SC maintenance, participants sought to always or daily support the patient to prevent illness and checked for the presence of lower limb edema. However, the daily monitoring of body weight and regular practice of physical activities were recommendations mentioned with low frequency. Daily body weight monitoring under the same conditions (time, with light clothes, on the scale) is an adherence strategy for recognizing signs and symptoms of clinical deterioration caused by HF; and regular physical activity practice is a behavior for promoting the functional capacity and quality of life of the patient^(22)^. Despite this, the findings of the present study confirm research in which it was identified that caregivers never/rarely or only sometimes recommended to the patient with HF to engage in physical activity (60.6%) or exercise (64.8%)^(17)^.

Regarding the management behavior of SC, caregivers supported the patient in reducing salt intake in the diet. Spouses, children, and siblings were indicated as social referents who exerted the most influence on salt reduction in people with HF^(23)^. In contrast, caregivers considered it unlikely or somewhat unlikely to guide the patient in reducing fluid intake. A similar finding was observed in a Spanish study^(24)^.

Although caregivers supported the patient in contacting the health team for guidance, they stated they did not recognize or were slow to identify the symptoms of HF. This difficulty may delay seeking health services in situations of clinical decompensation, potentially worsening adverse outcomes. Research conducted in England corroborates that the lack of information and knowledge generates insecurity, fear, and unpreparedness to decide when faced with disease exacerbations^(25)^. This strengthens the understanding that the perception of signs and interpretation of HF symptoms are key components for maintaining and managing SC. In this context, factors such as age and social support contribute to the early recognition of symptoms^(26)^.

Taking an extra diuretic was the variable with the lowest frequency among all items of the CC-SCHFI. In Brazil, this practice is not established in the pharmacological treatment among patients and caregivers, nor is it widely explored in the guidance provided by health professionals and nurses in interventions. It is assumed that caregivers do not feel secure in making decisions in response to changes in the patient’s symptoms. A similar result was found in a qualitative study conducted in Italy^(27)^.

Diuretics are widely used medications to control symptoms and reduce fluid overload^(28)^. In this sense, it becomes necessary for health professionals to include and expand this information. Moreover, institutional protocols for diuretic adjustments could be established to reinforce the management of treatment and prevent acute congestion episodes.

In the items that make up the sub-scale of confidence, caregivers showed much or extreme confidence to assess, follow, and manage the treatment of HF. Confidence reflects the caregiver’s capacity to assist the person with HF^(10)^. Although the median reached is below the cutoff point, this result envisions the possibility of improving caregivers’ participation in SC. Evidence indicates that the caregiver’s confidence in contributing to the patient’s SC is a predictive variable for SC management^(14)^. Based on findings like this, the need to incorporate strategies to increase the caregiver’s level of knowledge about HF, as well as to prepare and engage the caregiver in seeking positive outcomes for the patient, is reinforced.

In this study, no significant correlations were identified between age, education, and hours of care with the CC-SCHFI sub-scales. Contrary to these findings, research has shown that younger caregiver age and fewer hours of care are associated with their contribution to SC management^(17)^. Therefore, more research is necessary to clarify these issues.

Given the above, nurses must concentrate efforts to plan and test interventions aimed at SC behavior, considering cultural differences and access to health services, with the central focus of care on patients and their families, so that better health indicators and quality of life can be achieved. Supporting this proposition, a clinical trial to evaluate the efficacy of an educational multimedia intervention (textual resources, visuals, discussions, and telephone follow-up)-and thus improve self-efficacy, perceived control, and knowledge of HF by caregivers-showed satisfactory results in maintenance, management, and perceived control of caregivers contributing to SC^(29)^. In line with this, the American Heart Association emphasizes that the use of technology has the potential to increase the efficiency and effectiveness of care delivery in support of patient and caregiver health^(30)^.

### Study limitations

It should be noted that the cross-sectional design does not allow for establishing causality relationships, which requires caution in interpreting the results. Additionally, the self-reporting of variables may have been influenced by memory bias.

It is understood that SC behavior is influenced over time by the trajectory of HF. Therefore, to better understand the phenomenon, it is advisable to conduct longitudinal studies and bivariate tests, considering larger samples in different regions with the inclusion of new variables such as knowledge, dyad analysis (patient-caregiver), and demographic and psychosocial factors.

### Contributions to the Fields of Nursing, Health, or Public Policy

This study has implications for advancing knowledge in the health field, especially in nursing, as the results highlight the practices of caregivers in favor of SC, which serve as a starting point for developing and testing educational interventions aimed at caregivers and individuals with HF, an action still incipient in the national context. Thus, the findings can support the implementation of practices with the potential to provide disease knowledge, personalized follow-up, and evaluations of SC behavior patterns.

## CONCLUSIONS

This study assessed that the contribution of informal caregivers regarding maintenance, management, and confidence in heart failure self-care was inadequate. None of the scales reached the cutoff point ≥ 70 points. Therefore, health educational interventions are necessary to foster knowledge and skills in caregivers so that they can contribute effectively to self-care.
