# Supplementary material for: Contribution of informal caregivers to self-care in individuals with heart failure
Source: Rev Bras Enferm. 2024 Jul 29;77(3):e20230492. doi: 10.1590/0034-7167-2023-0492 (PMC11290721; doi:10.1590/0034-7167-2023-0492)
Supplement: Translation

# Translation: Contribuição de cuidadores informais para o autocuidado de pessoas com insuficiência cardíaca

**DOI:** 10.1590/0034-7167-2023-0492pt

**Authors:** Mailson Marques de Sousa, Adriana Meira Tiburtino Nepomuceno, Rayana Pereira Feitosa, Lara de Sá Neves Loureiro, Renan Alves Silva, Maria das Graças Melo Fernandes, Simone Helena dos Santos Oliveira

**Affiliations:** I Universidade Federal da Paraíba. João Pessoa, Paraíba, Brasil; II Hospital Universitário Lauro Wanderley. João Pessoa, Paraíba, Brasil; III Universidade Federal de Campina Grande. Cajazeiras, Paraíba, Brasil

**Keywords:** Insuficiência Cardíaca, Autocuidado, Cuidadores, Enfermagem, Cardiologia

## Abstract

**Objetivos:**

avaliar a contribuição de cuidadores informais para o autocuidado de pessoas com insuficiência cardíaca.

**Métodos:**

estudo transversal, realizado com 87 cuidadores, de março a outubro de 2022, na cidade João Pessoa/PB. A contribuição do cuidador foi avaliada por meio do instrumento *Caregiver Contribution to Self-Care of Heart Failure Index*. Escores ≥ 70 pontos indicam contribuição adequada. Os dados foram analisados por meio de estatística descritiva e correlação de Spearman.

**Resultados:**

eram do sexo feminino 81,6% da amostra. A mediana dos escores obtidos para as escalas de contribuição para o autocuidado foram: 63,3 para manutenção; 55,5 para manejo; e 66,6 para confiança. Os cuidadores nunca ou raramente recomendavam o monitoramento do peso corporal, a prática regular de exercícios físicos, o uso extra de diuréticos e a restrição de líquidos.

**Conclusões:**

os cuidadores informais apresentaram contribuição inadequada nos quesitos manutenção, manejo e confiança do autocuidado de pessoas com insuficiência cardíaca.

## INTRODUÇÃO

A insuficiência cardíaca (IC) é uma doença crônica, associada a índices expressivos de morbimortalidade, baixa qualidade de vida e custos financeiros elevados para os sistemas de saúde^(1)^. Estima-se que 64,34 milhões de pessoas no mundo possuam IC^(2)^. Projeções apontam que a prevalência de IC nos Estados Unidos deve aumentar de 5,8 milhões para 8,5 milhões, em 2030^(3)^. No Brasil, no triênio 2019-2021, foram autorizadas 533.055 internações hospitalares de pessoas com IC apenas no Sistema Único de Saúde, com taxa de mortalidade de 12,27% para o período^(4)^.

O autocuidado (AC) constitui estratégia fundamental para o seguimento terapêutico da IC. Trata-se de um processo de tomada de decisão naturalista que abrange a escolha de comportamentos assumidos pelos pacientes para manter a estabilidade fisiológica e responder aos sintomas da exacerbação da IC quando eles ocorrem^(5)^. Melhores níveis de autocuidado se relacionam com menores taxas de readmissão, melhor qualidade de vida e menor mortalidade^(6-7)^.

Estudos evidenciam que os comportamentos de AC em pessoas com IC são insatisfatórios^(8 9)^. Fatores como conhecimento, atitudes, habilidades, motivação, crenças, diferenças culturais e acesso a serviços de saúde estão associados^(5)^. Os comportamentos de AC em pessoas com IC, muitas vezes, são desenvolvidos com a contribuição de cuidadores informais (pessoas não remuneradas, com as quais o paciente possui relação preestabelecida como cônjuges, filhos, familiares ou amigos)^(10)^. Cerca de 45% a 70% dos pacientes com IC convivem com um familiar envolvido no cuidado^(11)^.

Na IC, a contribuição do cuidador para o AC é definida como o processo por meio do qual os cuidadores apoiam os pacientes na manutenção da estabilidade da doença, no monitoramento, na percepção e no gerenciamento dos sinais e sintomas, sendo mediado pela confiança em contribuir com AC dos pacientes sob seus cuidados^(10)^. Assim, os cuidadores informais auxiliam recomendando à pessoa com IC a verificação do peso corporal diário, o consumo de dieta com baixo teor de sódio, o controle da ingestão de líquidos, o uso de medicamentos prescritos, a prática de atividades físicas e vacinação. Fornecem apoio social, no monitoramento de sintomas e nas respostas às manifestações clínicas relacionadas à doença, quando elas ocorrem. Essa ajuda compreende a disponibilização de tempo, esforço e apoio em nome de outra pessoa que está realizando o AC nas ações de vida diária e auxílio na tomada de decisão^(10,12)^.

Estudo de revisão integrativa^(13)^ identificou que a maioria das pesquisas que avaliaram a contribuição do cuidador para o AC foram desenvolvidas em países da Europa e do Oriente Médio. Na América do Sul, há escassez de evidências, e não são claras as práticas realizadas pelos cuidadores em prol do AC dessa população. No Brasil, destaca-se investigação conduzida na Região Sudeste^(14)^, a qual evidenciou contribuição inadequada. Até o momento, não foram localizados estudos sobre a temática no Nordeste brasileiro. Dessa forma, justifica-se a importância de novas pesquisas para compreender o fenômeno e verificar a contribuição do cuidador na tomada de decisão para o AC e no gerenciamento da IC, a fim de minimizar hospitalizações indesejáveis, melhorar a qualidade de vida relacionada à saúde e projetar intervenções educativas factíveis na prática clínica.

## OBJETIVOS

Avaliar a contribuição de cuidadores informais para o autocuidado de pessoas com insuficiência cardíaca.

## MÉTODOS

### Aspectos éticos

O projeto de pesquisa foi aprovado pelo Comitê de Ética em Pesquisa da instituição proponente. Os participantes receberam informações verbais e escritas sobre o estudo e formalizaram a participação mediante a assinatura do Termo de Consentimento Livre e Esclarecido (TCLE).

### Desenho, período e local do estudo

Trata-se de um estudo transversal, no qual foram seguidas as recomendações do *Strengthening the Reporting of Observational Studies in Epidemiology* (STROBE). Foi desenvolvido em um ambulatório de cardiologia de um hospital universitário vinculado ao Sistema Único de Saúde, referência no atendimento de média e alta complexidade em cardiologia, localizado na cidade de João Pessoa, estado da Paraíba, Brasil. Nesse ambulatório, as pessoas com IC são referenciadas pela rede municipal de saúde para as consultas especializadas em cardiologia que ocorrem semanalmente. Na instituição selecionada, não há consulta de enfermagem nem grupos de apoio instituídos sob protocolo de *follow-up* para pessoas com IC. Entretanto, os cuidadores acompanham as consultas das pessoas com IC.

### População, amostra, critérios de inclusão e exclusão

A população do estudo foi constituída por cuidadores informais que acompanhavam pessoas com IC em seguimento ambulatorial da instituição. A amostra não probabilística foi selecionada por conveniência, no período de coleta de dados, entre março e outubro de 2022; e foi constituída de 87 cuidadores, designados pelos pacientes com IC como referência principal de cuidado.

Quanto aos critérios de elegibilidade, incluíram-se cuidadores informais com idade ≥ 18 anos, que estivessem cuidando dos pacientes por, no mínimo, três meses. Optou-se por considerar o período de três meses por causa de estudo prévio^(15)^ que identificou evolução nas ações de autocuidado em pessoas com IC. Os critérios de exclusão foram: cuidadores que apresentavam dificuldade de compreender o objetivo do estudo bem como de responder aos instrumentos de coleta de informações e cuidadores remunerados.

### Protocolo do estudo

Os dados foram coletados por meio de entrevista individual estruturada, em ambiente reservado, com o tempo médio de 30 minutos para cada entrevista. Uma equipe de pesquisa formada por três enfermeiras com experiência clínica em cardiologia foi treinada e compareceu ao ambulatório estando de posse da lista com os nomes dos pacientes agendados para consulta no dia. Essa lista foi extraída do sistema de agendamento interno, a fim de identificar aqueles pacientes que tinham cuidadores responsáveis e aqueles que atenderiam aos critérios de inclusão. Assim, antes ou depois da consulta médica, os cuidadores foram convidados a participar da pesquisa. Todos os que atenderam aos critérios de inclusão e foram convidados aceitaram o convite.

Utilizaram-se dos seguintes instrumentos para coleta de dados: a) formulário para caracterização do cuidador, contendo determinadas variáveis (idade, sexo, cor da pele autodeclarada, situação conjugal, escolaridade, renda familiar, situação de trabalho, relação do cuidador com o paciente, horas diárias de cuidado, acompanhamento nas consultas e participação em atividades de educação em saúde); b) *Caregiver Contribution to Self-Care of Heart Failure Index* (CC-SCHFI), versão traduzida e validada para o português do Brasil, para avaliar a contribuição do cuidador para o AC na IC^(16)^.

O CC-SCHFI compreende 22 itens distribuídos em três escalas: Contribuição do cuidador para manutenção do AC - dez itens (avalia os comportamentos do cuidador para monitorização dos sintomas e adesão às medidas terapêuticas para manutenção da estabilidade da IC); Contribuição do cuidador para o manejo do AC - seis itens (mensura os comportamentos do cuidador adotados para reconhecer sintomas de descompensação e intervir nos sintomas da IC); e Confiança do cuidador para contribuir com o AC - seis itens (analisa a capacidade do cuidador para incentivar o paciente a realizar o AC)^(16)^. Os autores do CC-SCHFI autorizaram o uso do instrumento, que demonstrou adequadas evidências de validade e confiabilidade na população brasileira^(16)^. Nessa investigação, o índice de confiabilidade das escalas do CC-SCHFI, avaliado por meio do alfa de Cronbach, foi 0,73 para a escala de manutenção do AC; 0,61 para a escala de manejo do AC; e 0,88 para a escala de confiança do AC.

As opções de resposta do CC-SCHFI variam em uma escala do tipo Likert de quatro pontos para cada uma das escalas. Para a de manutenção do AC, as respostas apresentam variação de “nunca/raramente” a “sempre/diariamente”; na escala de manejo do AC, variam de “improvável” a “muito provável”; e, para a escala relativa à confiança do cuidador para contribuir com o AC, as respostas vão de “nenhuma confiança” a “extrema confiança”. O escore total padronizado varia de 0 a 100; e cada subescala deve ser calculada e analisada separadamente. Escores ≥ 70 pontos indicam contribuição adequada do cuidador para o AC^(16)^.

### Análise dos resultados e estatística

Os dados foram organizados em planilha Excel e, em seguida, transportados para o software estatístico *Statistical Package for the Social Sciences* (SPSS), versão 22.0. Avaliou-se a normalidade dos dados pelo teste de Kolmogorov-Smirnov, que identificou distribuição assimétrica. Os dados foram analisados por meio de estatística descritiva. As variáveis categóricas foram expressas por frequências absolutas e relativas; e as variáveis numéricas, com mediana e intervalos interquartílicos 25 e 75.

Foi empregada a análise do coeficiente de correlação de Spearman entre os escores do CC SCHFI e variáveis quantitativas de interesse (idade, escolaridade e horas de cuidado) com base em evidências da literatura^(10,17)^ que destacam essas características dos cuidadores como fatores possíveis de influenciar a contribuição do AC na IC. Para classificar a força das correlações, adotou se o seguinte parâmetro: < 0,30, fraca magnitude; ≥ 0,30 e ≤ 0,50, moderada magnitude; > 0,50, forte magnitude^(18)^. O nível de significância adotado para as análises foi de *p* < 0,05.

## RESULTADOS

Quanto ao perfil dos cuidadores informais (n = 87), 81,6% eram do sexo feminino, com mediana de idade de 46 anos, 56,3% da cor parda, 71,3% casados, 29,9% autônomos, 56,3% com renda familiar de um salário mínimo, com escolaridade mediana de 8 anos. Quanto ao grau de parentesco do cuidador com o paciente, 75,9% tinham relações familiares, 41,4% eram filhas e 34,5% eram esposas, 24,1% não tinham grau de parentesco (vizinhos/amigos), 62,1% residiam com o paciente e dispensavam a mediana de 12 horas diárias de cuidado e 89,7% não participaram de atividades educativas relacionadas ao AC na insuficiência cardíaca.

A Tabela 1 apresenta a descrição dos escores da contribuição do cuidador para o autocuidado na IC. Verifica-se que nenhuma das escalas do CC-SCHFI alcançou o ponto de corte ≥ 70 pontos, considerado contribuição adequada para o AC.

**Tabela 1:** Escores do *Caregiver Contribution to Self-Care of Heart Failure Index*, João Pessoa, Paraíba, Brasil, 2022

Escalas	Mediana	IQ25-75^*^	Mínimo-Máximo
Manutenção do AC	63,3	53,3-73,3	17-100
Manejo do AC	55,5	44,4-72,2	11-89
Confiança do AC	66,6	55,5-77,7	11-100

* *Intervalo interquartílico 25 e 75; AC - autocuidado.*

A Tabela 2 apresenta a frequência das respostas por item para manutenção do AC do CC SCHFI. Ao considerar as contribuições dos cuidadores informais, verificou-se que estes nunca ou raramente recomendavam à pessoa com IC pesar-se diariamente (55,2%), realizar alguma atividade física (42,5%) e exercitar-se por 30 minutos (43,7%). Entretanto, apoiavam-na a comparecer às consultas com o médico ou enfermeiro (73,6%) e a usar sistemas de lembretes para tomar medicamentos (42,5%).

**Tabela 2:** Frequências de respostas dos itens para manutenção do autocuidado, João Pessoa, Paraíba, Brasil, 2022

Manutenção para o AC	*Nunca ou raramente*	*Às vezes*	*Frequentemente*	*Sempre ou diariamente*
Pesar-se diariamente	48 (55,2)	20 (23,0)	14 (16,1)	5 (5,7)
Verificar se os tornozelos estão inchados	13 (14,9)	15 (17,2)	37 (42,5)	22 (25,3)
Evitar ficar doente (p.ex., vacinar-se contra a gripe e evitar contato com pessoas doentes)	-	9 (10,3)	24 (27,6)	54 (62,1)
Realizar alguma atividade física	37 (42,5)	16 (18,4)	21 (24,1)	13 (14,9)
Comparecer a consultas com médico ou enfermeiro	-	6 (6,9)	17 (19,5)	64 (73,6)
Ingerir dieta com pouco sal	4 (4,6)	8 (9,2)	17 (19,5)	58 (66,7)
Exercitar-se por 30 minutos	38 (43,7)	16 (18,4)	21 (24,1)	12 (13,8)
Lembrar-se de tomar os remédios	4 (4,6)	11 (12,6)	16 (18,4)	56 (64,4)
Solicitar alimentos com pouco sal quando come fora ou visita alguém	5 (5,7)	9 (10,3)	32 (36,8)	41 (47,1)
Usar um sistema (caixa de comprimidos, lembretes etc.) que o(a) ajude a lembrar‑se dos medicamentos	21 (24,1)	14 (16,1)	15 (17,2)	37 (42,5)

*AC - autocuidado.*

No manejo do AC, os cuidadores informais relataram ser improvável recomendar tomar um diurético a mais (89,7%) ou reduzir o consumo de líquidos (31,0%). A maioria dos cuidadores recomendou diminuir o sal na dieta (63,2%) e entrar em contato imediatamente com o médico ou enfermeiro para solicitar orientação terapêutica e/ou de cuidados (79,3%) (Tabela 3).

**Tabela 3:** Frequências de respostas dos itens do manejo do autocuidado, João Pessoa, Paraíba, Brasil, 2022

Manejo do AC	*Não reconheci*	*Demorei/* *Demorei um pouco*	*Rapidamente*	*Imediatamente*
Se a pessoa de quem você cuida tem dificuldade para respirar ou inchaço nos tornozelos… Com que rapidez você reconheceu isso como um sintoma de insuficiência cardíaca?	12 (13,8)	24 (27,6)	30 (34,5)	21 (24,1)
Reduzir o sal na dieta	*Improvável* 4 (4,6)	*Um pouco provável* 4 (4,6)	*Provável* 24 (27,6)	*Muito provável* 55 (63,2)
Reduzir a ingestão de líquidos	27 (31,0)	12 (13,8)	18 (20,7)	30 (34,5)
Tomar um diurético a mais	78 (89,7)	4 (4,6)	2 (2,3)	3 (3,4)
Entrar em contato com o médico ou enfermeiro para solicitar orientação	-	-	18 (20,7)	69 (79,3)
Pense em uma medida que você tentou na última vez em que a pessoa de quem você cuida teve dificuldade para respirar ou inchaço nos tornozelos… Quanto você teve de certeza de que essa medida ajudou?	*Não tentei/Nenhuma* 8 (9,2)	*Um pouco de certeza* 17 (19,5)	*Certeza* 42 (48,3)	*Muita certeza* 20 (23,0)

*AC - autocuidado.*

Com relação à confiança para contribuir com o AC, os cuidadores sentiram muita ou extrema confiança em seguir o tratamento e menos confiança em fazer algo para aliviar os sintomas da IC ou reconhecer as alterações na saúde (Tabela 4).

**Tabela 4:** Frequências de respostas dos itens da confiança para contribuir com o autocuidado, João Pessoa, Paraíba, Brasil, 2022

Confiança para contribuir com o AC	*Nenhuma*	*Pouca*	*Muita*	*Extrema*
Prevenir sintomas de IC	6 (6,9)	14 (16,1)	50 (57,5)	17 (19,5)
Seguir as recomendações de tratamento	1 (1,1)	6 (6,9)	49 (56,3)	31 (35,6)
Avaliar a importância dos sintomas de IC	6 (6,9)	14 (16,1)	46 (52,9)	21 (24,1)
Reconhecer as alterações na saúde da pessoa de quem você cuida	3 (3,4)	11 (12,6)	46 (52,9)	27 (31,0)
Fazer algo que alivie os sintomas de IC	5 (5,7)	20 (23,0)	45 (51,7)	17 (19,5)
Avaliar o quanto uma medida funciona	5 (5,7)	18 (20,7)	46 (52,9)	18 (20,7)

*AC - autocuidado.*

A Tabela 5 apresenta os coeficientes de correlações de Spearman entre os escores do CC SCHFI e as variáveis dos cuidadores. Verificam-se correlações de fraca magnitude e não significantes.

**Tabela 5:** Coeficientes de correlações entre variáveis dos cuidadores e as escalas do *Caregiver Contribution to Self-Care of Heart Failure Index*, João Pessoa, Paraíba, Brasil, 2022

Variáveis	Manutenção do AC	Manejo do AC	Confiança do AC
Idade	-0,09	-0,14	0,01
Escolaridade	0,19	0,20	0,17
Horas de cuidado	-0,02	0,00	0,06

*AC - autocuidado.*

## DISCUSSÃO

Este estudo objetivou avaliar a contribuição do cuidador para o autocuidado na IC em uma capital do Nordeste do Brasil. Quanto ao perfil dos cuidadores informais, observou-se prevalência de mulheres, semelhante ao encontrado em estudo americano^(19)^ e nacional^(16)^. Corrobora esse dado o fato de as funções de gênero demandadas pela sociedade atribuírem às mulheres papéis socioculturais de proteção familiar com responsabilidades pelo exercício das atividades da vida diária, cuidados pessoais e emocionais, o que indica a relevante função delas no cuidado das pessoas, especialmente no contexto familiar. Além disso, também se verificou que os cuidadores entrevistados tinham mediana de idade inferior à observada em pesquisas^(19-20)^.

A baixa escolaridade e a ausência de participação em atividades educativas pelos cuidadores deste estudo constituem condições que podem interferir na contribuição do cuidador para o AC. Esses resultados são similares aos identificados em estudo qualitativo^(21)^, que mostrou, entre os desafios enfrentados pelos cuidadores no cuidado da pessoa com IC, o déficit de conhecimento básico sobre doença, medicamentos, além de falta de orientação adequada dos profissionais da equipe e da organização dos serviços de saúde. Outro estudo observou que os cuidadores realizavam cuidados com incerteza e conhecimento inadequado^(13)^. Do mesmo modo, os achados da presente pesquisa reforçam a necessidade de a equipe de referência viabilizar estratégias de educação em saúde para o cuidador e incorporá-lo ao atendimento clínico a fim de promover o desenvolvimento de aptidões para o desempenho de cuidados seguros.

Nesta investigação, embora a maioria dos cuidadores fossem membros da família, é provável que o conhecimento sobre a síndrome, as habilidades e o tempo disponibilizado para o cuidado tenham sido ineficientes para favorecer a manutenção do AC. A IC é uma doença crônica, com complexo regime terapêutico, que requer engajamento na mudança do estilo de vida e apoio social contínuo para incorporar as ações de AC^(5,7)^. Pesquisas apontam que a sobrecarga, o estresse e os sintomas de ansiedade e depressão prejudicam a contribuição do cuidador para o AC^(10,12-13)^. Diante desse achado, recomenda-se o desenvolvimento de novos estudos, a fim de explorar o grau de relação paciente-cuidador e o impacto da díade no comportamento de AC.

Constatou-se que a contribuição para o AC dada pelos cuidadores era inadequada, já que nenhuma das escalas (manutenção, manejo e confiança do AC) atingiu o ponto de corte ≥ 70 pontos. As pontuações alcançadas foram superiores aos escores obtidos em pesquisa realizada na Itália^(17)^ e similares aos do estudo conduzido em São Paulo^(14)^. Uma possível explicação é que os cuidadores necessitam de orientações direcionadas, tempo, apoio social e motivação para o exercício das atividades, especialmente para o manejo não farmacológico da pessoa com IC. Outro aspecto que deve ser considerado é a ausência de consultas de enfermagem específicas no seguimento dessa população no contexto local. Esses dados são relevantes para o planejamento de melhorias nos serviços de saúde frente às orientações prévias e para o cuidado usual fornecidas pelos profissionais da equipe multidisciplinar.

Com relação à análise descritiva dos itens que compõem o CC-SCHFI, na escala de contribuição do cuidador para manutenção do AC, os participantes procuravam sempre ou diariamente apoiar o paciente a fim de evitar que ele adoecesse e verificavam a presença de edema de membros inferiores. Contudo, a medida diária do monitoramento do peso corporal e a prática regular de atividades físicas foram recomendações mencionadas com baixa frequência. A verificação diária do peso corporal nas mesmas condições (horário, com roupas leves e balança) é uma estratégia de adesão para reconhecer sinais e sintomas de deterioração clínica ocasionada pela IC; e a prática regular de atividades físicas é um comportamento para promoção da capacidade funcional e qualidade de vida do paciente^(22)^. Apesar disso, os achados do presente estudo confirmam pesquisa na qual se identificou que os cuidadores nunca/raramente ou apenas às vezes recomendavam ao paciente com IC realizar atividade física (60,6%) ou exercício (64,8%)^(17)^.

Quanto ao comportamento de manejo do AC, os cuidadores apoiavam o paciente a reduzir o consumo de sal na dieta. Cônjuges, filhos e irmãos foram indicados como referentes sociais que exerciam maior influência na redução de sal em pessoas com IC^(23)^. Em contraponto, os cuidadores consideraram improvável ou pouco provável orientar o paciente sobre diminuir a ingestão de líquidos. Dado semelhante foi encontrado em estudo espanhol^(24)^.

Embora os cuidadores apoiassem o paciente a entrar em contato com a equipe de saúde para buscar orientações, eles afirmaram não reconhecer ou demorar a identificar os sintomas da IC. Essa dificuldade pode retardar a procura por serviços de saúde em situações de descompensação clínica, potencializando desfechos adversos. Pesquisa realizada na Inglaterra corrobora que a falta de informação e conhecimento gera insegurança, medo e despreparo para tomar decisões diante de exacerbações da doença^(25)^. Isso fortalece o entendimento de que a percepção dos sinais e a interpretação dos sintomas da IC são componentes-chave para manutenção e gerenciamento do AC. Nesse contexto, fatores como idade e apoio social contribuem para o reconhecimento precoce de sintomas^(26)^.

Destaca-se que tomar um diurético a mais foi a variável que apresentou a menor frequência entre todos os itens do CC-SCHFI. No Brasil, essa prática não é consolidada no tratamento medicamentoso entre pacientes e cuidadores, além de pouco explorada nas orientações fornecidas pelos profissionais de saúde e enfermeiros em intervenções. Supõe-se que os cuidadores não se sintam seguros para tomada de decisão diante de alterações nos sintomas do paciente. Resultado similar foi encontrado em estudo qualitativo realizado na Itália^(27)^.

Os diuréticos são medicamentos amplamente utilizados para controlar sintomas e reduzir a sobrecarga hídrica^(28)^. Nesse sentido, torna-se necessário que os profissionais de saúde incluam e ampliem essa informação. Além disso, protocolos institucionais para ajustes de diuréticos poderiam ser instituídos, com a finalidade de reforçar o manejo do tratamento e prevenir quadros agudos de congestão.

Nos itens que compõem a subescala de confiança, os cuidadores apresentaram muita ou extrema confiança para avaliar, seguir e gerenciar o tratamento da IC. A confiança reflete a capacidade do cuidador para ajudar a pessoa com IC^(10)^. Embora a mediana alcançada seja inferior ao ponto de corte, esse resultado vislumbra a possibilidade de melhorar a participação dos cuidadores no AC. Evidências apontam que a confiança do cuidador em contribuir para o AC do paciente constitui variável preditora para o manejo do AC^(14)^. Com base em achados como este, reforça-se a necessidade de incorporar estratégias para elevar o nível de conhecimento sobre a IC bem como aumentar o preparo e o engajamento do cuidador na busca de desfechos positivos para o paciente.

Neste estudo, não se identificaram correlações significantes entre idade, escolaridade e horas de cuidado e as subescalas do CC-SCHFI. Diferentemente desses achados, pesquisa evidenciou que a idade mais jovem do cuidador e menos horas de cuidado estão associadas com a contribuição deste para o manejo do AC^(17)^. Logo, mais pesquisas são necessárias para elucidar essas questões.

Diante do exposto, os enfermeiros devem concentrar esforços para planejar e testar intervenções voltadas ao comportamento do AC considerando as diferenças culturais e de acesso a serviços de saúde, tendo como objeto central do cuidado pacientes e seus familiares, de modo que sejam alcançados melhores indicadores em saúde e qualidade de vida. Corroborando essa proposição, ensaio clínico para avaliar a eficácia de uma intervenção educacional multimídia (recursos textuais, visuais, discussões e seguimento telefônico) - e, assim, melhorar a autoeficácia, o controle percebido e o conhecimento da IC de cuidadores - apresentou resultados satisfatórios na manutenção, no gerenciamento e controle percebido do cuidador para contribuir com o AC^(29)^. Nessa linha, a American Heart Association enfatiza que o uso de tecnologia apresenta potencial para aumentar a eficiência e a eficácia da prestação de cuidados em prol de apoiar a saúde de pacientes e cuidadores^(30)^.

### Limitações do estudo

Destaca-se que o desenho transversal não permite estabelecer relações de causalidade, o que demanda cautela na interpretação dos resultados. Além disso, o autorrelato das variáveis pode ter sido influenciado pelo viés de memória.

Entende-se que o comportamento de AC sofre influência ao longo do tempo da trajetória da IC. Portanto, para melhor compreensão do fenômeno, é recomendável a realização de estudos longitudinais e testes bivariados, contemplando amostras maiores, em diferentes regiões, com a inclusão de novas variáveis, como conhecimento, análise da díade (paciente-cuidador) e fatores demográficos e psicossociais.

### Contribuições para a área da Enfermagem, Saúde ou Política Pública

Este estudo traz implicações para o avanço do conhecimento na área da saúde, em especial para a enfermagem, pois os resultados evidenciam as práticas dos cuidadores em favor do AC, as quais constituem ponto de partida para elaborar e testar intervenções educativas voltadas a cuidadores e pessoas com IC, o que ainda é uma ação incipiente no contexto nacional. Dessa forma, os resultados podem subsidiar a implementação de práticas com potencial de fornecer conhecimento sobre a doença, seguimento personalizado e avaliações de padrões de comportamento de AC.

## CONCLUSÕES

Este estudo avaliou que a contribuição de cuidadores informais para manutenção, manejo e confiança em relação ao autocuidado na insuficiência cardíaca foi inadequada. Nenhuma das escalas atingiu o ponto de corte ≥ 70 pontos. Portanto, intervenções educativas em saúde são necessárias para fomentar o conhecimento e habilidades do cuidador, de modo que ele possa contribuir com o autocuidado.
